# Longitudinal risk of death, hospitalizations for atrial fibrillation, and cardiovascular events following catheter ablation of atrial fibrillation: a cohort study

**DOI:** 10.1093/ehjqcco/qcac024

**Published:** 2022-06-14

**Authors:** Linh Ngo, Richard Woodman, Russell Denman, Tomos E Walters, Ian A Yang, Isuru Ranasinghe

**Affiliations:** Greater Brisbane Clinical School, Faculty of Medicine, The University of Queensland, Northside Clinical Unit, The Prince Charles Hospital, Chermside, 4032, QLD, Australia; Department of Cardiology, The Prince Charles Hospital, Chermside, 4032, QLD, Australia; Flinders Centre for Epidemiology and Biostatistics, College of Medicine and Public Health, Flinders University, Bedford Park, 5042, SA, Australia; Department of Cardiology, The Prince Charles Hospital, Chermside, 4032, QLD, Australia; Cardiology, St Vincent's Private Hospital Northside, Chermside, 4032, QLD, Australia; Greater Brisbane Clinical School, Faculty of Medicine, The University of Queensland, Northside Clinical Unit, The Prince Charles Hospital, Chermside, 4032, QLD, Australia; Department of Thoracic Medicine, The Prince Charles Hospital, Chermside, 4032, QLD, Australia; Greater Brisbane Clinical School, Faculty of Medicine, The University of Queensland, Northside Clinical Unit, The Prince Charles Hospital, Chermside, 4032, QLD, Australia; Department of Cardiology, The Prince Charles Hospital, Chermside, 4032, QLD, Australia

**Keywords:** Catheter ablation, Atrial fibrillation, Long-term outcomes

## Abstract

**Aims:**

Population studies reporting contemporary long-term outcomes following catheter ablation of atrial fibrillation (AF) are sparse.

We evaluated long-term clinical outcomes following AF ablation and examined variation in outcomes by age, sex, and the presence of heart failure.

**Methods and results:**

We identified 30 601 unique patients (mean age 62.7 ± 11.8 years, 30.0% female) undergoing AF ablation from 2008 to 2017 in Australia and New Zealand using nationwide hospitalization data. The primary outcomes were all-cause mortality and rehospitalizations for AF or flutter, repeat AF ablation, and cardioversion. Secondary outcomes were rehospitalizations for other cardiovascular events. During 124 858.7 person-years of follow-up, 1900 patients died (incidence rate 1.5/100 person-years) with a survival probability of 93.0% (95% confidence interval (CI) 92.6–93.4%) by 5 years and 84.0% (95% CI 82.4–85.5%) by 10 years. Rehospitalizations for AF or flutter (13.3/100 person-years), repeat ablation (5.9/100 person-years), and cardioversion (4.5/100 person-years) were common, with respective cumulative incidence of 49.4% (95% CI 48.4–50.4%), 28.1% (95% CI 27.2–29.0%), and 24.4% (95% CI 21.5–27.5%) at 10 years post-ablation. Rehospitalizations for stroke (0.7/100 person-years), heart failure (1.1/100 person-years), acute myocardial infarction (0.4/100 person-years), syncope (0.6/100 person-years), other arrhythmias (2.5/100 person-years), and new cardiac device implantation (2.0/100 person-years) occurred less frequently. Elderly patients and those with comorbid heart failure had worse survival but were less likely to undergo repeat ablation, while long-term outcomes were comparable between the sexes.

**Conclusion:**

Patients undergoing AF ablations had good long-term survival, a low incidence of rehospitalizations for stroke or heart failure, and about half remained free of rehospitalizations for AF or flutter, including for repeat AF ablation, or cardioversion.

## Graphical Abstract

**Graphical Abstract:**
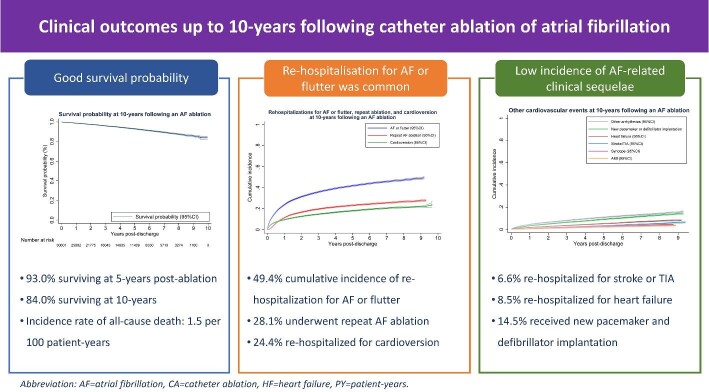

## Introduction

Nearly one in four men and one in five women after the age of 40 will have atrial fibrillation (AF), the most common sustained heart rhythm disorder.^1,2^ Besides the high prevalence, AF is associated with a higher risk of adverse outcomes, including death, stroke, heart failure, and acute myocardial infarction.^3^ While medical therapy has low efficacy in restoring normal sinus rhythm,^4^ catheter ablation is a more effective option to terminate AF and improve symptoms.^5,6^ Moreover, AF patients with comorbid heart failure who undergo ablation experience a survival benefit in addition to symptoms relief when compared with medical therapy.^7^

Although the benefits of catheter ablation in maintaining sinus rhythm have been well established in clinical trials, long-term clinical sequelae of patients undergoing AF ablation in actual clinical practice are less well known. Published studies mostly report arrhythmia-free survival rate and are often derived from selected populations and experienced ablation centres,^6,8,9^ making their results less representative of outcomes in mainstream clinical practice. Furthermore, while the frequently used definition of arrhythmia occurrence (AF lasting >30 s)^10^ is useful in clinical trial settings, less is known about the incidence of clinically meaningful episodes such as those warranting hospitalization or requiring repeat ablation or cardioversion. Moreover, data are lacking about the risk of other equally important cardiovascular outcomes such as mortality, stroke, and heart failure, which is crucial for patients and clinicians seeking to better understand long-term outcomes of AF ablation. Population studies can provide unbiased estimates of these long-term outcomes, yet existing literature is sparse and limited by short follow-up time (up to 1 year)^11–13^ or a focus on specific populations.^14–16^

Accordingly, we used population-wide data to examine long-term outcomes of patients undergoing AF ablation in Australia and New Zealand (ANZ) from 2008 to 2017. Specifically, we evaluated the longitudinal risk of all-cause mortality, rehospitalizations for AF or flutter, repeat AF ablation, cardioversion, and other cardiovascular events, including hospitalizations for stroke or transient ischaemic attack (TIA) and heart failure. We also examined how the primary outcomes varied by age, sex, and the presence of comorbid heart failure as this subgroup of AF patients has been shown to experience survival benefits with catheter ablation compared with medical therapy.^7^

## Methods

### Data source

We used hospitalization data from all public and most (80%) private hospitals recorded in the Admitted Patient Collection (APC) from each state and territory in Australia and the equivalent New Zealand National Minimum Dataset (Hospital Events). Data were missing from private hospitals in New Zealand and the Australian states of South Australia, Tasmania, and Northern Territory, whose total population accounted for <10% of the total Australian population. A standard set of variables is recorded for each admission (both inpatient and outpatient visits), including patient demographic characteristics, the primary diagnosis and up to 50 secondary diagnoses, all procedures performed, and the patient's status at discharge. In both countries, diagnoses are coded using the International Classification of Diseases, 10th Revision, Australian Modification (ICD-10-AM), and procedures were coded using the Australian Classification of Health Interventions (ACHI). Accuracy of diagnosis and procedure coding is reported to exceed 85% when compared with medical records.^17,18^

In Australia, each encounter was linked with subsequent records within the APC and to each region's Birth, Death, and Marriages Registry by using probabilistic matching using multiple patient identifiers with reported accuracy exceeding 99%.^19^ In New Zealand, all patient records are linked nationally using a unique National Health Index number, and all deaths are recorded in the National Health Index Sociodemographic Profile. These linkages allowed the capture of rehospitalizations to any hospital within each region and all deaths occurring in hospital or in the community.

### Study cohort

The use of administrative data to identify patients undergoing AF ablations has been described previously.^20,21^ In brief, we included patients aged ≥18 years hospitalized with a primary diagnosis of AF and a procedure code for catheter ablation from 2008 to 2017 and excluded those who (1) had secondary diagnoses of other arrhythmias to ensure that the ablation was solely for AF; (2) had a previous or current cardiac device implantation; (3) had open (surgical) ablation; and (4) were discharged against medical advice (see the supplementary material online, *Table S1* for all ICD-10-AM and ACHI codes used to identify the study cohort). The identification of AF ablation in administrative data has been validated to be highly accurate (100% specificity and 87.3% sensitivity).^22^ For patients with multiple admissions during the study period, the first episode was considered the index hospitalization, with all subsequent events considered an outcome (rehospitalization for AF).

### Study outcomes

The primary outcomes were (1) all-cause mortality and (2) rehospitalizations for atrial arrhythmias (AF or flutter), repeat AF ablation, and cardioversion. Secondary outcomes included relevant cardiovascular events, including hospitalizations for stroke or TIA, heart failure, acute myocardial infarction, syncope, arrhythmias other than AF or flutter (bradycardia and tachycardia, all types of heart block), and new cardiac device (pacemaker or defibrillator) implantation (see the Supplementary material online, *Table S1* for more details).

### Statistical analysis

We presented data as frequencies and percentages for categorical variables and mean ± standard deviation (SD) or median and interquartile range (IQR) for continuous variables. Differences between groups were evaluated using *χ^2^* or Fisher's exact test for categorical variables and Student’s *t*-test or Mann–Whitney *U* test for continuous variables where appropriate. For patients who were rehospitalized multiple times, only the first episode was counted. Comorbidities were derived using the Condition Categories system, that groups selected secondary diagnoses from the index hospitalization and all diagnoses (both primary and secondary) from hospitalizations in the previous 12 months into 180 meaningful clinical conditions.^23^

To estimate the incidence rate, which reflects the number of new events that occurred in a given period, we divided the number of events by the patient time at risk and reported results as the number of events per 100 person-years (PY). Patients were considered at risk until they died, experienced a non-fatal outcome, or survived until the end of the study period (1 January 2018). We used the Kaplan–Meier method to estimate the survival probability and reported results as percentages with the respective 95% confidence interval (CI). For non-fatal outcomes, we estimated the cumulative incidence, which reflects the proportion of patients experiencing the event over a given period by using Fine and Gray's method of competing risk survival analysis (subdistribution hazard) with death being the competing event.^24^

To examine variation in outcomes by age, sex, and comorbid heart failure, we used Cox regression survival analysis to adjust for baseline differences in patient and procedural characteristics. Simple Cox regression analysis was used with the outcome of death, while for non-fatal outcomes, separate competing risk models using Fine and Gray's method were developed with death being the competing event.^25^ Results are reported as hazard ratio (HR) for mortality or subdistribution HR (sHR) for non-fatal outcomes with the corresponding 95% CI. Candidate variables considered for these models included patient demographic characteristics (age, sex, and presenting region), procedural characteristics (elective admission, ablation of both atria, and ablation in a private hospital), and various cardiovascular and non-cardiovascular comorbidities that may be associated with long-term outcomes.

All analyses were performed using Stata version 16.0 ( StataCorp LLC, College Station, TX) and a two-tailed *P*-value < 0.05 was considered statistically significant. This study was approved by the respective Human Research Ethics Committees of each state and territory in Australia. Data from New Zealand were obtained under a data user agreement with the New Zealand Ministry of Health. A waiver of informed consent was provided for the use of deidentified data.

## Results

**Figure 1:**
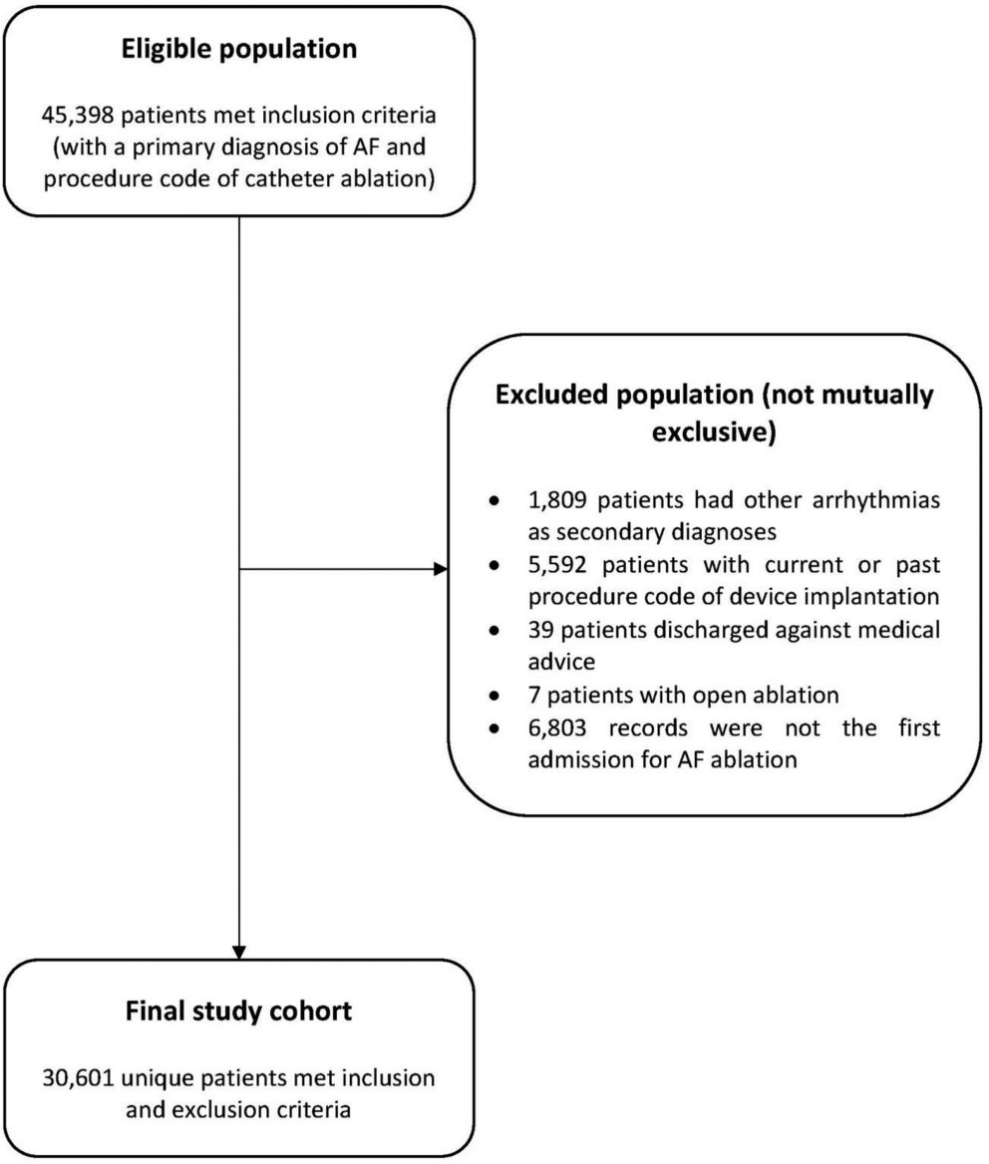
Patient selection flow diagram. AF, atrial fibrillation.

From 2008 to 2017, 45 398 patients were hospitalized with a primary diagnosis of AF and a procedure code for catheter ablation, of whom 30 601 unique patients met selection criteria and were included in the final study cohort (*Figure 1*).

### Cohort characteristics

The mean age was 62.7 (±11.8) years with 46.1% aged ≥65 years and the cohort was predominantly (70.0%) male (*Table 1*). The median length of stay was 1.0 day (IQR 1.0–2.0 days) and 94.0% of AF ablations were performed during an elective (planned) hospitalization. Nearly half of the study cohort (49.1%) had been hospitalized with a primary diagnosis of AF or flutter in the previous year, but rates of comorbidities were generally low, with hypertension (13.4%) and diabetes (10.1%) being the most common cardiac and non-cardiac comorbidities, respectively. The median estimated CHA_2_DS_2_-VASc score (thromboembolic risk score for AF patients in which a point each is given for the presence of congestive heart failure [C], hypertension [H], age 65-74 years old [A], diabetes [D], vascular disease (VASc) and female sex and 2 points each are given for age ≥75 years old and history of stroke [S]) was 1 (IQR 0–2) with 90.1% with a score ≤2.

**Table 1:** Baseline characteristics of the study cohort

		All-cause mortality			Rehospitalizations for AF or flutter		
Variables	Overall (*N* = 30 601) *n* (%)	Survived (*N* = 28 701) *n* (%)	Died (*N* = 1900) *n* (%)	*P* value	Not rehospitalized (*N* = 19 479) *n* (%)	Rehospitalized (*N* = 11 114) *n* (%)	*P-* value
Demographic characteristics							
Age (mean ± SD)	62.7 ± 11.8	62.0 ± 11.5	72.4 ± 10.8	<0.001	63.3 ± 12.1	61.5 ± 11.0	<0.001
Age group							
18–34	642 (2.1)	631 (2.2)	11 (0.6)	<0.001	427 (2.2)	215 (1.9)	<0.001
35–49	3316 (10.8)	3261 (11.4)	55 (2.9)	—	1993 (10.2)	1323 (11.9)	—
50–64	12 540 (41.0)	12 203 (42.5)	337 (17.7)	—	7536 (38.7)	5002 (45.0)	—
65–79	12 270 (40.1)	11 278 (39.3)	992 (52.2)	—	8053 (41.3)	4212 (37.9)	—
≥80	1833 (6.0)	1328 (4.6)	505 (26.6)	—	1470 (7.6)	362 (3.3)	—
Female (%)	9180 (30.0)	8588 (29.9)	592 (31.2)	0.255	5825 (29.9)	3351 (30.2)	0.650
Median length of stay (IQR)	1.0 (1.0–2.0)	1.0 (1.0–2.0)	1.0 (1.0–3.0)	<0.001	1.0 (1.0–2.0)	1.0 (1.0–2.0)	<0.001
Presenting region							
NZ	2572 (8.4)	2439 (8.5)	133 (7.0)	<0.001	1700 (8.7)	871 (7.8)	<0.001
ACT/NSW	8203 (26.8)	7672 (26.7)	531 (28.0)	—	5226 (26.8)	2974 (26.8)	—
SA/NT	2130 (7.0)	1932 (6.7)	198 (10.4)	—	1388 (7.1)	741 (6.7)	—
QLD	6152 (20.1)	5765 (20.1)	387 (20.4)	—	3687 (18.9)	2465 (22.2)	—
TAS	0 (0.0)	0 (0.0)	0 (0.0)	—	0 (0.0)	0 (0.0)	—
VIC	7433 (24.3)	7035 (24.5)	398 (21.0)	—	5121 (26.3)	2309 (20.8)	—
WA	4111 (13.4)	3858 (13.4)	253 (13.3)	—	2357 (12.1)	1754 (15.8)	—
Elective (scheduled) procedure	28 755 (94.0)	27 089 (94.4)	1666 (87.7)	<0.001	18 270 (93.8)	10 478 (94.3)	0.087
Private hospital	19 272 (63.0)	18 208 (63.4)	1064 (56.0)	<0.001	12 158 (62.4)	7111 (64.0)	0.006
CHA_2_DS_2_-VASc score^a^ (median, IQR)	1 (0-2)	1 (0-1)	2 (1-3)	<0.001	1 (0-2)	1 (0-1)	<0.001
0 (*n*, %)	13 012 (42.5)	12 818 (44.7)	194 (10.2)	<0.001	7916 (40.6)	5095 (45.8)	<0.001
1 (*n*, %)	9236 (30.2)	8807 (30.7)	429 (22.6)	—	5803 (29.8)	3430 (30.9)	—
≥2 (*n*, %)	8353 (27.3)	7076 (24.7)	1277 (67.2)	—	5760 (29.6)	2589 (23.3)	—
Cardiac history							
Hypertension	4098 (13.4)	3518 (12.3)	580 (30.5)	<0.001	2520 (12.9)	1575 (14.2)	0.002
Heart failure	3123 (10.2)	2574 (9.0)	549 (28.9)	<0.001	2046 (10.5)	1073 (9.7)	0.018
Valvular and rheumatic heart disease	1319 (4.3)	1149 (4.0)	170 (9.0)	<0.001	784 (4.0)	535 (4.8)	0.001
Coronary artery disease	3294 (10.8)	2858 (10.0)	436 (23.0)	<0.001	2136 (11.0)	1155 (10.4)	0.120
Vascular disease	514 (1.7)	426 (1.5)	88 (4.6)	<0.001	353 (1.8)	161 (1.5)	0.017
History of hospitalization for AF or flutter	15 035 (49.1)	14 117 (49.9)	918 (48.3)	0.462	8861 (45.5)	6171 (55.5)	<0.001
Non-cardiac comorbidities							
Diabetes mellitus	3101 (10.1)	2780 (9.7)	321 (16.9)	<0.001	2221 (11.4)	879 (7.9)	<0.001
Chronic lung diseases	1365 (4.5)	1060 (3.7)	305 (16.1)	<0.001	924 (4.7)	439 (4.0)	0.001
Chronic kidney disease	1072 (3.5)	811 (2.8)	261 (13.7)	<0.001	790 (4.1)	281 (2.5)	<0.001
Stroke or TIA	425 (1.4)	374 (1.3)	51 (2.7)	<0.001	261 (1.3)	164 (1.5)	0.329
Haematological disorders	1369 (4.5)	1086 (3.8)	283 (14.9)	<0.001	863 (4.4)	505 (4.5)	0.644
Pneumonia	667 (2.2)	511 (1.8)	156 (8.2)	<0.001	477 (2.5)	188 (1.7)	<0.001
Musculoskeletal and connective tissue disorders	2214 (7.2)	1981 (6.9)	233 (12.3)	<0.001	1379 (7.1)	833 (7.5)	0.177
Dementia and senility	55 (0.2)	41 (0.1)	14 (0.7)	<0.001	44 (0.2)	11 (0.1)	0.012
Major cancer	229 (0.8)	148 (0.5)	81 (4.3)	<0.001	182 (0.9)	47 (0.4)	<0.001
End-stage liver disease	39 (0.1)	30 (0.1)	9 (0.5)	0.001	27 (0.1)	12 (0.1)	0.470
Drug or alcohol abuse, psychosis, or dependence	458 (1.5)	383 (1.3)	75 (4.0)	<0.001	304 (1.6)	153 (1.4)	0.202
Psychiatric disorders	447 (1.5)	376 (1.3)	71 (3.7)	<0.001	291 (1.5)	156 (1.4)	0.527
Neurological disorders and paralysis	326 (1.1)	275 (1.0)	51 (2.7)	<0.001	211 (1.1)	115 (1.0)	0.691
Skin ulcers	99 (0.3)	55 (0.2)	44 (2.3)	<0.001	79 (0.4)	20 (0.2)	0.001
Urinary tract disorders and incontinence	1283 (4.2)	1081 (3.8)	202 (10.6)	<0.001	874 (4.5)	408 (3.7)	0.001

Major cancer includes metastatic cancer and acute leukaemia; lung, upper digestive tract, and other severe cancers; and lymphatic, head and neck, brain, and other major cancers. Drug-related disorders include drug or alcohol psychosis, drug or alcohol abuse with or without dependence.SD, standard deviation; IQR, interquartile range; NZ, New Zealand; ACT, Australian Capital Territory; NSW, New South Wales; SA, South Australia; NT, Northern Territory; QLD, Queensland; TAS, Tasmania; VIC, Victoria; WA, Western Australia; and TIA, transient ischaemic attack; ^a^CHA_2_DS_2_-VASc score is a score used to evaluate risk of experiencing thromboembolic events of AF patients in which a point each is given for the presence of congestive heart failure (C), hypertension (H), age 6574 years old (A), diabetes (D), vascular disease (VASc) and female sex and two points each are given for age> = 75 years old and history of stroke (S). The total score ranges from 0 to 9 with the higher the score, the higher the risk.^33^

During 124 858.7 person-years of follow-up (median follow-up time of 3.8 years, IQR: 1.7–6.2 years), 1900 patients died. Compared with those who survived, deceased patients were older (72.4 vs. 62.0 years), less likely to have an elective index hospitalization (87.7% vs. 94.4%), and had a higher frequency of comorbidities such as hypertension (30.5% vs. 12.3%), heart failure (28.9% vs. 9.0%), coronary artery disease (23.0% vs. 10.0%), diabetes (16.9% vs. 9.7%), and a higher CHA_2_DS_2_-VASc score (median score of 2 vs. 1) (all *P*-values < 0.001).

A total of 30 593 patients survived the index hospitalizations, among whom 11 114 were rehospitalized for AF or flutter during follow-up. Rehospitalized patients were younger (61.5 vs. 63.3 years), had higher rate of hypertension (14.2% vs. 12.9%), and were more likely to be hospitalized for AF or flutter in the previous year (55.5% vs. 45.5%) compared with those not rehospitalized.

### Long-term clinical outcomes following atrial fibrillation ablation

#### All-cause mortality

**Figure 2:**
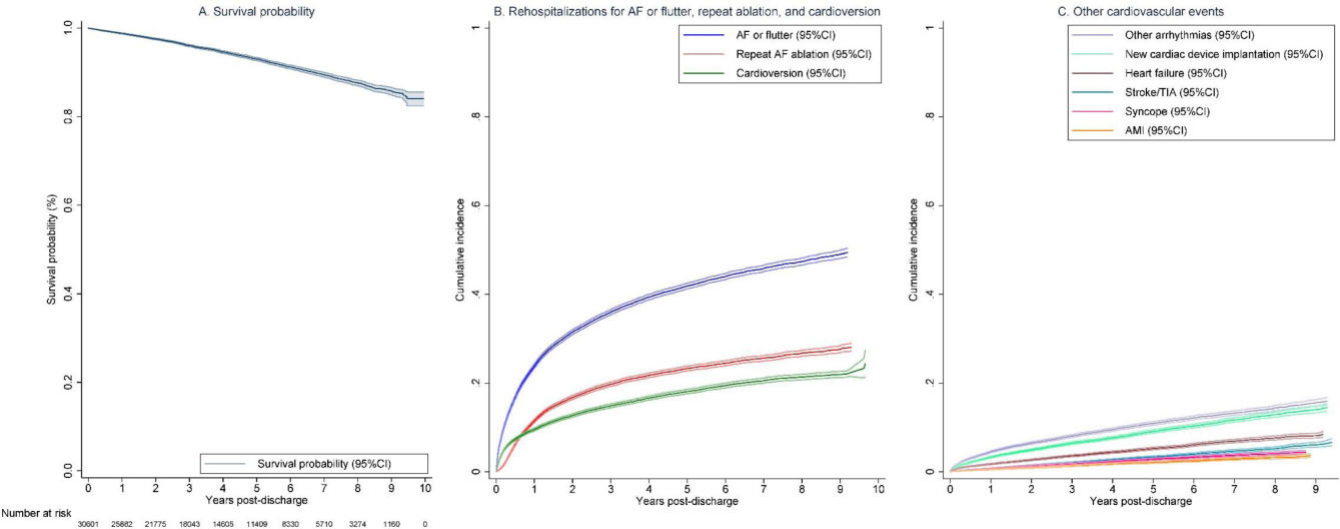
Long-term outcomes following catheter ablation of atrial fibrillation. (*A*) Survival probability following catheter ablation of atrial fibrillation. (*B*) Cumulative incidence of rehospitalizations for AF or flutter, and related procedures (repeat AF ablation and cardioversion). (*C*) Cumulative incidence of rehospitalizations for other cardiovascular events. AF, atrial fibrillation; AFL, atrial flutter; AMI, acute myocardial infarction; CI, confidence interval; and TIA, transient ischaemic attack.

The overall incidence rate of all-cause death was 1.5/100 PY (*Table 2* and *Figure 2*), which increased from 1.2/100 PY in the first year to 1.5/100 PY in 1–5 years and reached 2.0/100 PY at 5–10 years after the index ablation. This corresponded to a survival probability of 98.8% (95% CI 98.6–98.9%) at 1 year, 93.0% (95% CI 92.6–93.4%) at 5 years, and 84.0% (95% CI 82.4–85.5%) at 10-year, respectively.

**Table 2:** Long-term outcomes of patients undergoing catheter ablation of atrial fibrillation

Outcomes	Total number of patients *N* (incidence rate)	1-year incidence rate *N* (incidence rate)	1–5 years incidence rate *N* (incidence rate)	5–10 years incidence rate *N* (incidence rate)	Cumulative incidence (95% CI)
Death	1900 (1.5)	348 (1.2)	1069 (1.5)	483 (2.0)	16.0% (14.5%–17.6%)
Rehospitalizations for AF or flutter	11 114 (13.3)	6923 (29.0)	3706 (7.8)	485 (3.9)	49.4% (48.4%–50.4%)
Acute AF/AFL rehospitalizations	5148 (4.8)	2760 (10.5)	2029 (3.3)	359 (2.0)	24.8% (24.0%–25.7%)
Repeat AF ablation or cardioversion					
Repeat AF ablation	6001 (5.9)	3251 (12.2)	2458 (4.2)	292 (1.7)	28.1% (27.2%–29.0%)
Cardioversion of AF/AFL	4811 (4.5)	2792 (10.6)	1725 (2.8)	294 (1.6)	24.4% (21.5%–27.5%)
Rehospitalizations for other cardiovascular events					
Stroke/TIA	883 (0.7)	212 (0.8)	495 (0.7)	176 (0.8)	6.6% (5.8%–7.5%)
Heart failure	1385 (1.1)	505 (1.8)	674 (0.9)	206 (0.9)	8.5% (7.9%–9.2%)
Acute myocardial infarction	549 (0.4)	142 (0.5)	305 (0.4)	102 (0.4)	3.7% (3.3%–4.2%)
Syncope	716 (0.6)	209 (0.7)	400 (0.6)	107 (0.5)	4.3% (4.0%–4.7%)
Other arrhythmias (tachycardia and bradycardia, heart block)	2858 (2.5)	1304 (4.8)	1246 (1.8)	308 (1.5)	15.9% (15.0%–16.7%)
New pacemaker or defibrillator implantation	2393 (2.0)	951 (3.4)	1111 (1.6)	331 (1.6)	14.5% (13.6%–15.3%)

AF, atrial fibrillation; AFL, atrial flutter; TIA, transient ischaemic attack; SVT, supraventricular tachycardia; and VT, ventricular tachycardia.

#### Rehospitalization for atrial fibrillation or flutter

A total of 11 114 patients experienced at least one acute (unplanned) or elective (planned) rehospitalization for AF or flutter (incidence rate 13.3/100 PY). The incidence rate peaked during the first year (29.0/100 PY) and rapidly declined to 7.8/100 PY and 3.9/100 PY at 1–5 years and 5–10 years post-ablation, respectively. The cumulative incidence of rehospitalizations for atrial arrhythmias was 23.8% (95% CI 23.4–24.3%) at 1-year, and 49.4% (95% CI 48.4–50.4%) at 10 years. Of these 11 114 patients, 5148 (46.3%) had an acute (unplanned) admission for AF or flutter with an overall incidence rate of 4.8/100 PY and a cumulative incidence of 24.8% (95% CI 24.0–25.7%).

#### Repeat atrial fibrillation ablation and cardioversion

Among the 11 114 patients rehospitalized for AF or flutter, a subset of 6001 patients underwent repeat AF ablation (incidence rate 5.9/100 PY) and 4811 patients received cardioversion (incidence rate 4.5/100 PY). The incidence rate for repeat ablation was highest in the first year (12.2/100 PY), and then decreased to 1.7/100 PY in years 5–10 post ablation. The 10-year cumulative incidence was 28.1%, (95% CI 27.2–29.0%). Similarly, there was a rapid decline in the incidence rate of cardioversion for AF or flutter from 10.6/100 PY in the first year to 1.6/100 PY in years 5–10 post-ablation with an overall cumulative incidence of 24.4% (95% CI 21.5–27.5%).

#### Rehospitalizations for other cardiovascular events

Overall, the incidence rates of other cardiovascular events were low. Specifically, rehospitalizations for stroke or TIA occurred at an incidence rate of 0.7/100 PY with a cumulative incidence of 6.6% (95% CI 5.8–7.5%). Similarly, the incidence rate for heart failure (1.1/100 PY) and acute myocardial infarction (0.4/100 PY) was low with a cumulative incidence of 8.5% (95% CI 7.9–9.2%) and 3.7% (95% CI 3.3–4.2%), respectively. Rehospitalizations for syncope occurred with an incidence rate of 0.6/100 PY with a cumulative incidence of 4.3% (95% CI 4.0–4.7%). The estimates for rehospitalizations for arrythmias other than AF or flutter were 2.5/100 PY and 15.9% (95% CI 15.0–16.7%) respectively. A total of 14.5% (13.6–15.3%) of patients received either a pacemaker or defibrillator (incidence rate of 2.0/100 PY) during the 10-year period. The cumulative incidence was higher among patients with comorbid heart failure (19.8%, 95% CI 17.6–22.0%) compared with those without (13.9%, 95% CI 13.0–14.8%).

### Variation in primary outcomes by age, sex, and comorbid heart failure

**Figure 3:**
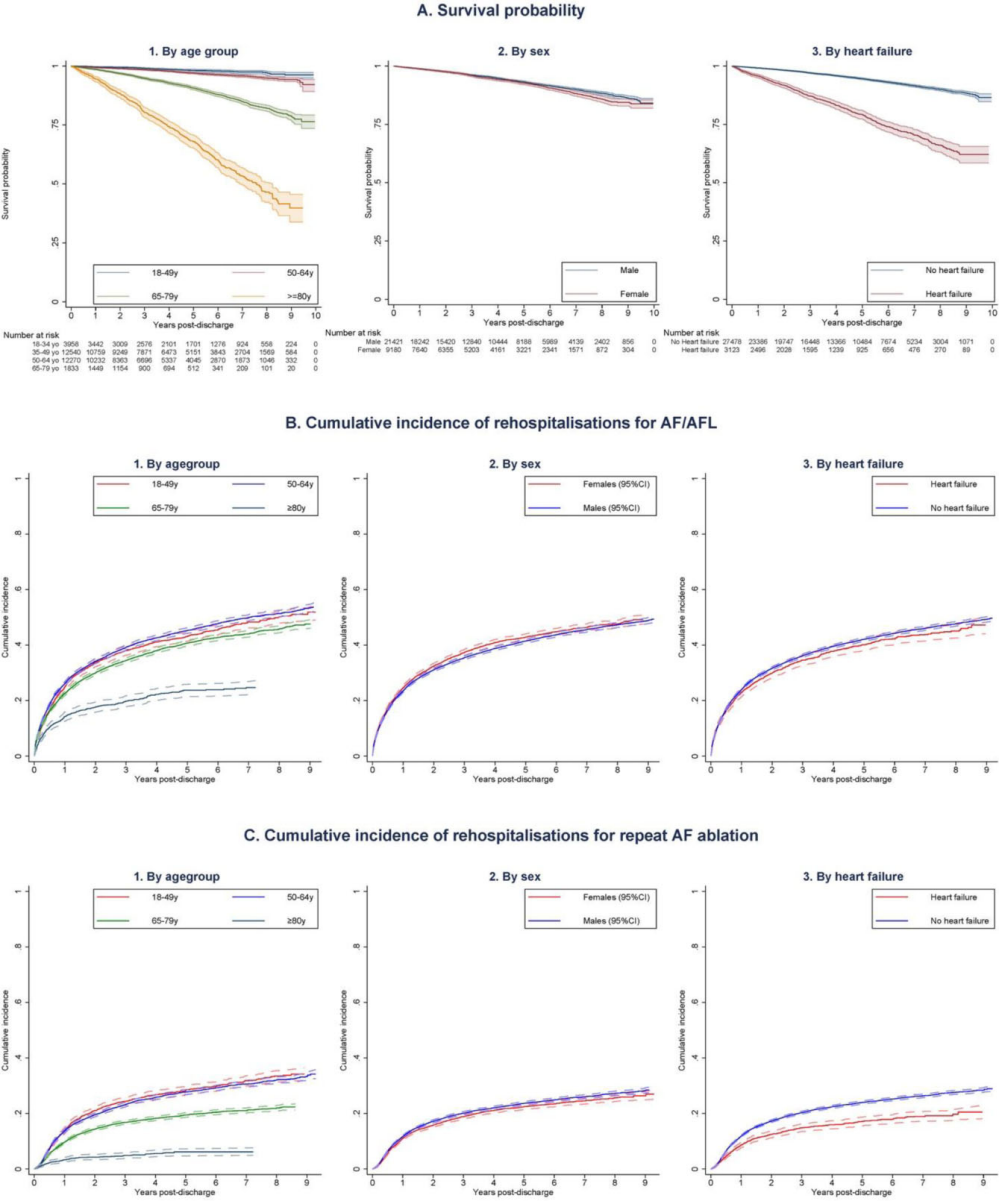
Long-term outcomes following catheter ablation of atrial fibrillation by age, sex, and comorbid heart failure. (*A*) Long-term survival probability following catheter ablation of atrial fibrillation based on (1) Age group. (2) Sex (3) The presence of comorbid heart failure. (*B*): Long-term risk of rehospitalizations for recurrent atrial arrhythmias (atrial fibrillation or flutter) following catheter ablation of atrial fibrillation based on (1) Age group, (2) Sex (3) The presence of comorbid heart failure. (*C*) Long-term risk of repeat AF ablation following catheter ablation of atrial fibrillation based on (1) Age group, (2) Sex, (3) The presence of comorbid heart failure. AF, atrial fibrillation; AFL, atrial flutter; and y, years old.

The unadjusted analysis showed survival probability declined in elderly patients and those with comorbid heart failure but was comparable between sexes (*Figure 3A*). Long term cumulative incidence of rehospitalizations for AF or flutter was lower among patients ≥80 years but was comparable between subgroups regardless of sex or the presence of heart failure (*Figure 3B*). Similarly, cumulative incidence of repeat ablation was lowest in the oldest age group and comparable between sex but was lower in patients with comorbid heart failure compared with those without heart failure (*Figure 3C*).

After adjusting for differences in other patient characteristics, female sex was associated with better survival (HR 0.83, 95% CI 0.75–0.92) while older age (HR for each decade increase in age: 2.40, 95% CI 2.28–2.52) and heart failure (HR 1.90, 95% CI 1.69–2.14) was associated with worse long-term survival (Supplementary material online, *Figure S1*). After accounting for the competing risk of death, older age was associated with lower adjusted hazard of rehospitalizations for AF or flutter (sHR 0.93, 95% CI 0.91–0.94) but higher hazard of repeat AF ablation (sHR 1.35, 95% CI 1.16–1.57). Female sex was associated with higher hazard of being rehospitalized for AF or flutter (sHR 1.06, 95% CI 1.02–1.11) but hazard of repeat ablation was similar between sex (sHR 0.95, 95% CI 0.69–1.31), while heart failure had no significant relationship with these outcomes (Supplementary material online, *Figures S2* and *S3*).

## Discussion

We found that patients undergoing AF ablations in ANZ had a good long-term clinical outcome with a high survival probability and a low incidence of clinical sequelae of AF such as rehospitalizations for stroke or TIA, heart failure, and acute myocardial infarction. Furthermore, about half of the patients did not experience rehospitalization for AF or flutter, including for repeat AF ablation or cardioversion. Collectively, these findings suggest a good prognosis after AF ablation but also suggest the need for additional measures to reduce the burden of AF as nearly 50% of patients required hospitalization for AF or flutter in the 10 years post-ablation.

Population studies play an important role in providing outcome data in clinical practice but existing studies primarily focus on short-term outcomes, typically up to 1 year post-ablation,^11–13^ while long-term outcome data mostly come from Asian countries^14,15^ or selected populations such as those who had cardioversion before ablation.^16^ Our study provided long-term outcomes from an unselected, contemporary cohort in ANZ, irrespective of age or payer. Our population was older than that in Korea and Taiwan (mean age of 51–57 years)^14,15^ but comparable with Denmark (mean age 65.5 years)^16^ and North America (60.0–65.3 years).^11–13^ Despite these differences, the incidence rate of death was consistent among studies (1.1/100 PY)^14–16^ but nearly double what is reported in large, multicentre clinical trials such as the Catheter Ablation vs. Antiarrhythmic Drug Therapy for Atrial Fibrillation (CABANA) trial (0.63/100 PY).^6^ Similarly, rates of rehospitalizations for stroke or TIA (0.5–0.7/100 patient-years)^14–16^ and heart failure (0.7–1.2/100 patient-years)^14–16^ were consistent among population studies but higher than those reported in the CABANA trial (0.01/100 PY for disabling stroke). Collectively, patients undergoing AF ablation in clinical practice appear to have a relatively low incidence of death and hospitalizations for stroke or heart failure, although the risks are greater than those reported in clinical trials.

We also extend the literature by reporting the longitudinal risk of hospitalization for recurrent AF or flutter and the need for repeat intervention-outcomes of critical importance that are frequently sought by patients and clinicians but rarely reported by other population studies.^14–16^ Although the definition of AF recurrence as an episode lasting >30 s is commonly used in clinical trials and recommended by management guidelines,^10^ measuring events that require hospital admission might better reflect the direct burden these patients face. Indeed, Terricabras and colleagues have shown that regardless of AF recurrence status, catheter ablation significantly reduces the AF burden and improves quality of life, suggesting that AF burden might be a more relevant outcome measure for patients undergoing AF ablation.^26^ Encouragingly, we found that just over 50% of patients were free of hospital admission for AF or flutter at 10 years post-ablation, and the incidence during the first year was only 23.8%, significantly lower than the 49.1% rate in the year preceding the ablation. Given the near 50% incidence of arrhythmia recurrence (defined as AF >30 s)^10^ at four years post-ablation reported in the CABANA trial,^6^ our finding (39.4% patients rehospitalized for AF or flutter at 4 years) suggests that 80% of these recurrences are likely severe enough to warrant rehospitalization. We also found that 28.1% of our patients required at least one repeat ablation, which is lower than the cumulative incidence (57.6%) reported in another population study.^16^ Nevertheless, the incidence rate of repeat AF ablation in our cohort was more than double that of the CABANA trial (5.9 vs. 2.6/100 patient-years),^8^ suggesting that in clinical practice, patients may require more frequent ablation to achieve outcomes comparable with the trial setting.

Another important observation is a comparable adjusted likelihood of repeat ablation in patients with heart failure despite the higher mortality risk in this subgroup. The literature has consistently shown better outcomes in heart failure patients undergoing AF ablations^27^ with the Catheter Ablation versus Standard Conventional Therapy in Patients with Left Ventricular Dysfunction and Atrial Fibrillation (CASTLE-AF) trial of AF ablation in those with severe heart failure (ejection fraction ≤35%) reporting a nearly 50% lower hazard of death compared with medical therapy.^7^ More importantly, this trial had a higher incidence of repeat ablation than that in our study (24.5% at 3.15 years vs. 14.8% at 3 years).^7^ This raises the possibility that AF ablation may be underutilized in patients with heart failure who might benefit most from the procedure.

Our findings provide important prognostic information for patients and clinicians who seek to be better informed about the long-term clinical outcomes of ablation. These data are reassuring as the incidence rates of untoward outcomes such as death, stroke or TIA, heart failure or acute myocardial infarction after ablation were relatively low. Nevertheless, nearly 50% of patients required hospitalization for AF or flutter and repeat ablation, suggesting that additional measures are required to minimize the AF burden. There are several strategies proven to minimize AF recurrence and improve ablation outcomes such as weight loss,^28^ reducing alcohol consumption,^29^ and optimal management of AF risk factors like comorbid hypertension, diabetes, and sleep apnoea.^30^ Systematic implementation of these strategies may further improve ablation outcomes, decrease the disease burden, and reduce the need of hospital admission or repeat procedures.

Several limitations should be considered when interpreting our results. First, we used routinely collected administrative data that are generally less granular than those collected specifically for research purposes. However, validation studies have shown good accuracy of coded data,^17,18^ linkages of health records,^19^ and identification of AF ablation in administrative data.^22^ Although the algorithm to identify AF ablation in administrative data may not capture all procedures as the sensitivity is not 100%, this approach has 100% specificity and has been widely used by other studies using hospitalization data.^31,32^ Second, we could not estimate the rate of atrial arrhythmia recurrence (defined as any atrial arrhythmia lasting >30 s^10^) as not all recurrences required hospital admission. Visits to the emergency department due to AF or flutter or other cardiovascular diseases after ablation that did not lead to hospitalization were also not counted. Instead, we focused on AF or flutter hospitalizations and other events that required hospital admission—events that are most concerning for patients and clinicians and have the greatest impact on health care resources. Third, patients undergoing AF ablation in Australia and New Zealand were relatively young and had low rates of comorbidities and therefore, our results may not reflect the outcomes of AF ablations in older and sicker patients. Fourth, this study did not seek to compare outcomes of patients undergoing ablation with those of patients who did not as many AF patients may present to general practitioners (not recorded in hospitalization data), while all AF ablations are performed in-hospital. Fifth, the inherent differences in study design and patient selection criteria must be considered when comparing our results with those in clinical trials.^6,7^ Nevertheless, such a comparison was helpful to better understand the outcomes of AF ablation in clinical practice. Finally, several variables that may influence long-term outcomes were not collected in these data sets and were therefore not adjusted for, such as type of AF, type of ablation energy or lesions performed, operator experience, cardiac function, and medications used.

## Conclusions

Patients undergoing catheter ablation of atrial fibrillation have a good long-term prognosis with 84.0% surviving by 10 years and relatively low incidence of sequelae such as stroke and heart failure. Furthermore, about half of the patients remained free of rehospitalizations for AF or flutter, repeat ablation, or cardioversion. Nevertheless, additional measures such as weight loss, alcohol abstinence, and better management of comorbidities are necessary to reduce the residual burden of AF post-ablation.

## Supplementary Material

qcac024_Supplemental_File

## Data availability

The data underlying this article were provided by a third party, including the Data Custodian Units of each state and territory in Australia, under ethics approval and the New Zealand Ministry of Health via a data user agreement. The data can be accessed upon request to the third party.

## References

[1] James SL , Abate D, Abate KH, Abay SM, Abbafati C, Abbasi N et al. Global, regional, and national incidence, prevalence, and years lived with disability for 354 diseases and injuries for 195 countries and territories, 1990–2017: a systematic analysis for the Global Burden of Disease study 2017. Lancet 2018;392:1789–1858. 10.1016/S0140-6736(18)32279-7 PMC6227754 30496104

[2] Lloyd-Jones DM , Wang TJ, Leip EP, Larson MG, Levy D, Vasan RS et al. Lifetime risk for development of atrial fibrillation: the Framingham Heart study. Circulation 2004;110:1042–1046. 15313941 10.1161/01.CIR.0000140263.20897.42

[3] Staerk L , Sherer JA, Ko D, Benjamin EJ, Helm RH. Atrial fibrillation: epidemiology, pathophysiology, and clinical outcomes. Circ Res 2017;120:1501–1517. 28450367 10.1161/CIRCRESAHA.117.309732 PMC5500874

[4] Calkins H , Reynolds MR, Spector P, Sondhi M, Xu Y, Martin A et al. Treatment of atrial fibrillation with antiarrhythmic drugs or radiofrequency ablation: two systematic literature reviews and meta-analyses. Circ Arrhythm Electrophysiol 2009;2:349–361. 19808490 10.1161/CIRCEP.108.824789

[5] Pappone C , Augello G, Sala S, Gugliotta F, Vicedomini G, Gulletta S et al. A randomized trial of circumferential pulmonary vein ablation versus antiarrhythmic drug therapy in paroxysmal atrial fibrillation: the APAF study. J Am Coll Cardiol 2006;48:2340–2347. 17161267 10.1016/j.jacc.2006.08.037

[6] Packer DL , Mark DB, Robb RA, Monahan KH, Bahnson TD, Poole JE et al. Effect of catheter ablation vs. antiarrhythmic drug therapy on mortality, stroke, bleeding, and cardiac arrest among patients with atrial fibrillation: the CABANA randomized clinical trial. JAMA 2019;321:1261–1274. 30874766 10.1001/jama.2019.0693 PMC6450284

[7] Marrouche NF , Brachmann J, Andresen D, Siebels J, Boersma L, Jordaens L et al. Catheter ablation for atrial fibrillation with heart failure. N Engl J Med 2018;378:417–427. 29385358 10.1056/NEJMoa1707855

[8] Nielsen JC , Johannessen A, Raatikainen P, Hindricks G, Walfridsson H, Pehrson SM et al. Long-term efficacy of catheter ablation as first-line therapy for paroxysmal atrial fibrillation: 5-year outcome in a randomised clinical trial. Heart 2017;103:368–376. 27566295 10.1136/heartjnl-2016-309781

[9] Scaglione M , Gallo C, Battaglia A, Sardi D, Gaido L, Anselmino M et al. Long-term progression from paroxysmal to permanent atrial fibrillation following transcatheter ablation in a large single-center experience. Heart Rhythm 2014;11:777–782. 24561164 10.1016/j.hrthm.2014.02.018

[10] Calkins H , Hindricks G, Cappato R, Kim YH, Saad EB, Aguinaga L et al. 2017 HRS/EHRA/ECAS/APHRS/SOLAECE expert consensus statement on catheter and surgical ablation of atrial fibrillation: executive summary. J Arrhythm 2017;33:369–409. 29021841 10.1016/j.joa.2017.08.001 PMC5634725

[11] Patil N , Arora S, Davis L, Akoum NW, Chung MK, Sridhar AR et al. Incidence and predictors of 30-day acute cerebrovascular accidents post atrial fibrillation catheter ablation (from the Nationwide Readmissions Database. Am J Cardiol 2021;138: 61–65. 33058801 10.1016/j.amjcard.2020.10.020

[12] Guo J , Nayak HM, Besser SA, Beaser A, Aziz Z, Broman M et al. Impact of atrial fibrillation ablation on recurrent hospitalization: a nationwide cohort study. JACC Clin Electrophysiol 2019;5:330–339. 30898236 10.1016/j.jacep.2018.10.015

[13] Arora S , Lahewala S, Tripathi B, Mehta V, Kumar V, Chandramohan D et al. Causes and predictors of readmission in patients with atrial fibrillation undergoing catheter ablation: a national population-based cohort study. J Am Heart Assoc 2018;7:e009294. 29907655 10.1161/JAHA.118.009294 PMC6220533

[14] Yang PS , Sung JH, Jang E, Yu HT, Kim TH, Uhm JS et al. Catheter ablation improves mortality and other outcomes in real-world patients with atrial fibrillation. J Am Heart Assoc 2020;9:e015740. 32427022 10.1161/JAHA.119.015740 PMC7429005

[15] Chang CH , Lin JW, Chiu FC, Caffrey JL, Wu LC, Lai MS. Effect of radiofrequency catheter ablation for atrial fibrillation on morbidity and mortality: a nationwide cohort study and propensity score analysis. Circ Arrhythm Electrophysiol 2014;7:76–82. 24446025 10.1161/CIRCEP.113.000597

[16] Modin D , Claggett B, Gislason G, Hansen ML, Worck R, Johannessen A et al. Catheter ablation for atrial fibrillation is associated with lower incidence of heart failure and death. Europace 2020;22:74–83. 31595294 10.1093/europace/euz264

[17] Henderson T , Shepheard J, Sundararajan V. Quality of diagnosis and procedure coding in ICD-10 administrative data. Med Care 2006;44:1011–1019. 17063133 10.1097/01.mlr.0000228018.48783.34

[18] Briffa T , Hung J, Knuiman M, McQuillan B, Chew DP, Eikelboom J et al. Trends in incidence and prevalence of hospitalization for atrial fibrillation and associated mortality in Western Australia, 1995–2010. Int J Cardiol 2016;208, 19–25. 10.1016/j.ijcard.2016.01.196 26826625

[19] Holman CD , Bass AJ, Rouse IL, Hobbs MS. Population-based linkage of health records in Western Australia: development of a health services research linked database. Aust N Z J Public Health 1999;23:453–459. 10575763 10.1111/j.1467-842x.1999.tb01297.x

[20] Ngo L , Ali A, Ganesan A, Woodman R, Adams R, Ranasinghe I. Gender differences in complications following catheter ablation of atrial fibrillation. Eur Heart J Qual Care Clin Outcomes 2021;7:458–467. 33963402 10.1093/ehjqcco/qcab035

[21] Ngo L , Ali A, Ganesan A, Woodman RJ, Adams R, Ranasinghe I. Utilisation and safety of catheter ablation of atrial fibrillation in public and private sector hospitals. BMC Health Serv Res 2021;21:883. 34454482 10.1186/s12913-021-06874-7 PMC8400841

[22] Singh SM , Webster L, Calzavara A, Wijeysundera HC. Validation of algorithms to identify invasive electrophysiology procedures using administrative data in Ontario, Canada. Med Care 2017;55:e44–e50. 25415560 10.1097/MLR.0000000000000274

[23] Pope GC , Kautter J, Ellis RP, Ash AS, Ayanian JZ, Lezzoni LI et al. Risk adjustment of Medicare capitation payments using the CMS-HCC model. Health Care Financ Rev 2004;25:119–141. 15493448 PMC4194896

[24] Fine JP , Gray RJ. A proportional hazards model for the subdistribution of a competing risk. J Am Statist Assoc 1999;94:496–509.

[25] Dignam JJ , Zhang Q, Kocherginsky M. The use and interpretation of competing risks regression models. Clin Cancer Res 2012;18:2301–2308. 22282466 10.1158/1078-0432.CCR-11-2097 PMC3328633

[26] Terricabras M , Mantovan R, Jiang CY, Betts TR, Chen J, Deisenhofer I et al. Association between quality of life and procedural outcome after catheter ablation for atrial fibrillation: a secondary analysis of a randomized clinical trial. JAMA Netw Open 2020;3:e2025473. 33275151 10.1001/jamanetworkopen.2020.25473 PMC7718606

[27] Turagam MK , Garg J, Whang W, Sartori S, Koruth JS, Miller MA et al. Catheter ablation of atrial fibrillation in patients with heart failure: a meta-analysis of randomized controlled trials. Ann Intern Med 2019;170:41–50. 30583296 10.7326/M18-0992

[28] Peigh G , Wasserlauf J, Vogel K, Kaplan RM, Pfenniger A, Marks D et al. Impact of pre-ablation weight loss on the success of catheter ablation for atrial fibrillation. J Cardiovasc Electrophysiol 2021;32:2097–2104. 34191371 10.1111/jce.15141 PMC9305992

[29] Takahashi Y , Nitta J, Kobori A, Sakamoto Y, Nagata Y, Tanimoto K et al. Alcohol consumption reduction and clinical outcomes of catheter ablation for atrial fibrillation. Circ Arrhythm Electrophysiol 2021;14:e009770. 33999699 10.1161/CIRCEP.121.009770

[30] Pathak RK , Middeldorp ME, Lau DH, Mehta AB, Mahajan R, Twomey D et al. Aggressive risk factor reduction study for atrial fibrillation and implications for the outcome of ablation: the ARREST-AF cohort study. J Am Coll Cardiol 2014;64:2222–2231. 25456757 10.1016/j.jacc.2014.09.028

[31] Deshmukh A , Patel NJ, Pant S, Shah N, Chothani A, Mehta K et al. In-hospital complications associated with catheter ablation of atrial fibrillation in the United States between 2000 and 2010: analysis of 93 ,801 procedures. Circulation 2013;128:2104–2112. 24061087 10.1161/CIRCULATIONAHA.113.003862

[32] Tripathi B , Arora S, Kumar V, Abdelrahman M, Lahewala S, Dave M et al. Temporal trends of in-hospital complications associated with catheter ablation of atrial fibrillation in the United States: an update from Nationwide Inpatient Sample database (2011–2014). J Cardiovasc Electrophysiol 2018;29:715–724. 10.1111/jce.13471 29478273

[33] Hindricks G , Potpara T, Dagres N, Arbelo E, Bax JJ, Blomström-Lundqvist C et al. 2020 ESC Guidelines for the diagnosis and management of atrial fibrillation developed in collaboration with the European Association for Cardio Thoracic Surgery (EACTS): the Task Force for the diagnosis and management of atrial fibrillation of the European Society of Cardiology (ESC) developed with the special contribution of the European Heart Rhythm Association (EHRA) of the ESC. Eur Heart J 2021;42:373–498. 32860505 10.1093/eurheartj/ehaa612

