# Supplementary material for: Longitudinal risk of death, hospitalizations for atrial fibrillation, and cardiovascular events following catheter ablation of atrial fibrillation: a cohort study
Source: Eur Heart J Qual Care Clin Outcomes. 2022 Jun 14;9(2):150–60. doi: 10.1093/ehjqcco/qcac024 (PMC9972809; doi:10.1093/ehjqcco/qcac024)
Supplement: qcac024_Supplemental_File [file qcac024_supplemental_file.docx]

**Supplemental Appendix**

**Manuscript title: Long-term outcomes of patients undergoing catheter ablation of atrial fibrillation: A bi-national cohort study**

Linh Ngo, Richard Woodman, Russell Denman, Tomos E. Walters, Ian A. Yang, Isuru Ranasinghe

**Supplemental Table**

**Supplemental Table S1:** Diagnosis and procedure codes used in this study

**Supplemental Figures**

**Supplemental Figure S1:** Risk factors of long-term death following catheter ablation of atrial fibrillation

**Supplemental Figure S2:** Risk factors of re-hospitalisation for recurrent atrial arrhythmias (atrial fibrillation or flutter) following catheter ablation of atrial fibrillation

**Supplemental Figure S3:** Risk factors of repeat ablation following catheter ablation of atrial fibrillation

**Supplemental Table S1: Diagnosis and procedure codes used in this study**

| **DISEASE/PROCEDURE** | **ICD10-AM/ACHI codes** |
| --- | --- |
| Atrial fibrillation | I48, I48.0, I48.1, I48.2, I48.9 |
| Atrial flutter | I48.3, I48.4 |
| Pre-excitation syndrome | I45.6 |
| Supra-ventricular tachycardia | I47.1 |
| Ventricular tachycardia | I47.2, I49.0 |
| Premature beats | I49.1, I49.2, I49.3, I49.4 |
| Arrhythmias other than AF or flutter | I45*, I47*, I49*, R00*, I44* |
| Presence of a cardiac device | Z95.0 |
| Stroke | I60*, I61*, I62*, I63*, I64* |
| Transient ischemic attack | G45* |
| Heart failure | I110, I130, I132, I50, I500, I509, U822 |
| Acute myocardial infarction | I21* |
| Syncope | R55 |
| Catheter ablation | 38287-01, 38287-02, 38290-01 |
| Pacemaker implantation | 38353-00 |
| Cardiac defibrillator implantation | 38393-00 |
| Open ablation | 38287-03, 38287-04, 38290-02 |

Footnote: ACHI = Australian Classification of Health Interventions; ICD10-AM = International Classification of Diseases, 10^th^ Revision, Australian Modification. *All individual codes are included.

**Figure S1: Risk factors of long-term death following catheter ablation of atrial fibrillation**


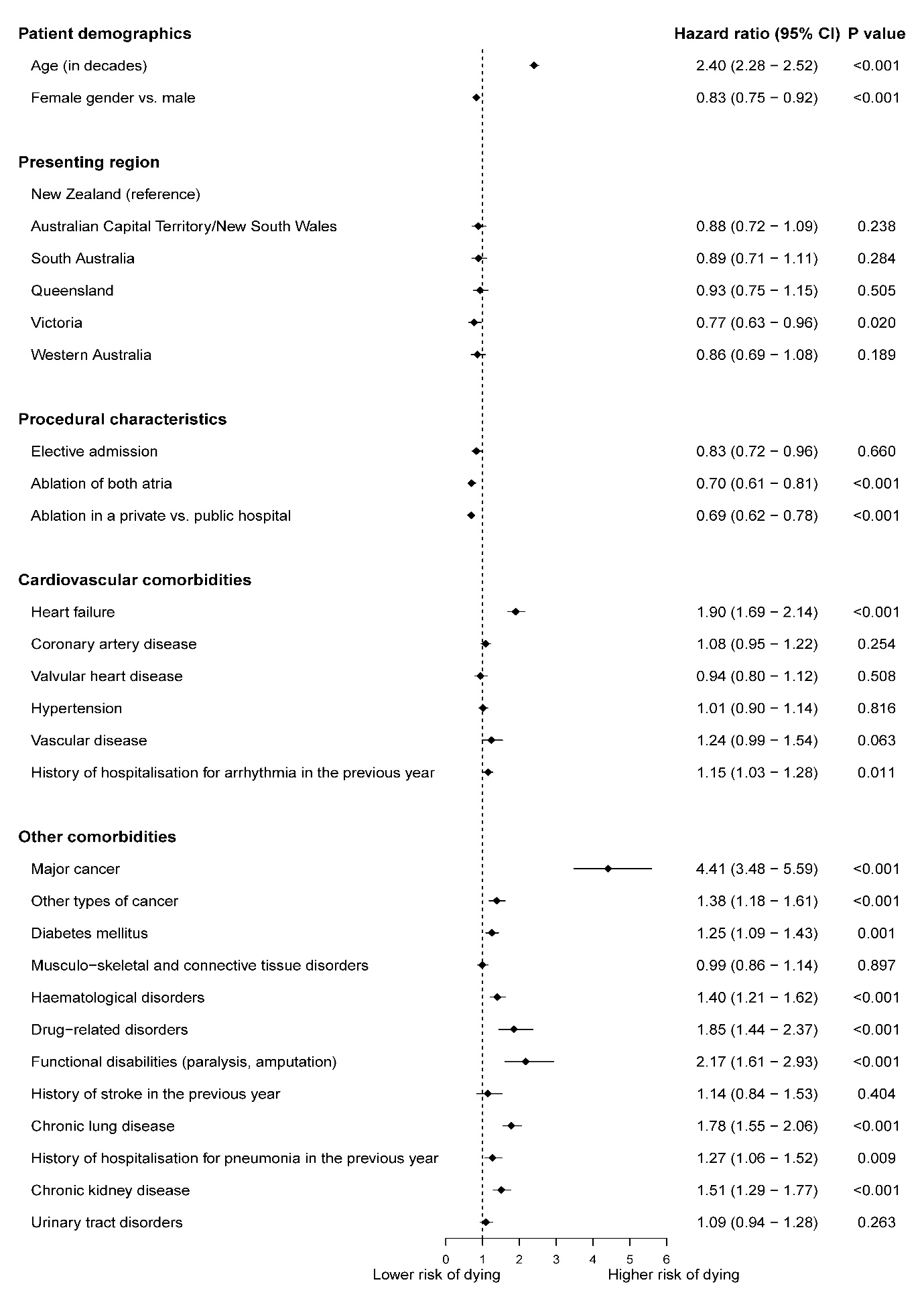


**Footnote:** Major cancer includes metastatic cancer and acute leukemia; lung, upper digestive tract, and other severe cancers; and lymphatic, head and neck, brain, and other major cancers. Drug-related disorders includes drug or alcohol psychosis, drug or alcohol abuse with or without dependence.

**Figure S2: Risk factors of re-hospitalisation for recurrent atrial arrhythmias (atrial fibrillation or flutter) following catheter ablation of atrial fibrillation**


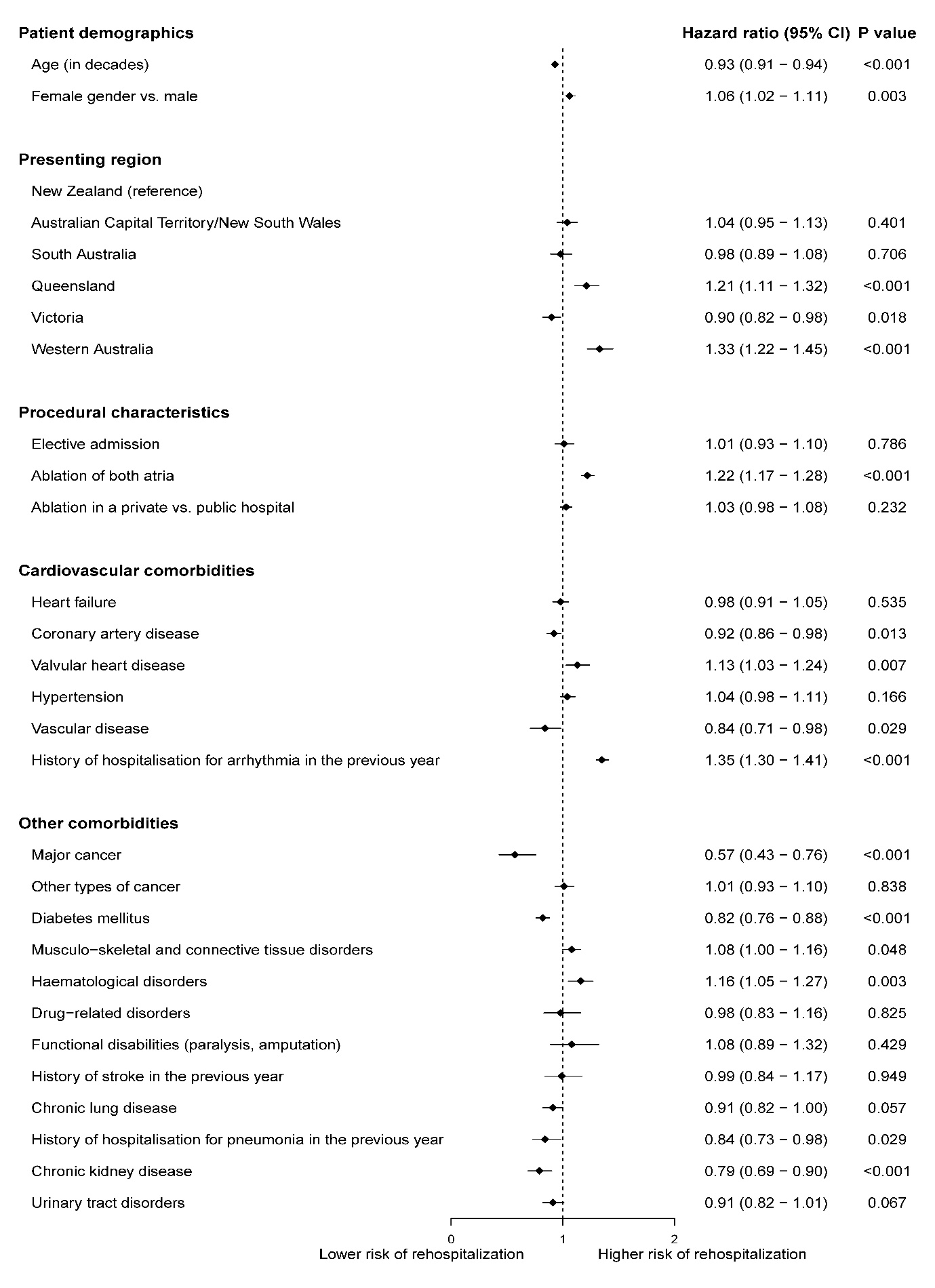


**Footnote:** Major cancer includes metastatic cancer and acute leukemia; lung, upper digestive tract, and other severe cancers; and lymphatic, head and neck, brain, and other major cancers. Drug-related disorders includes drug or alcohol psychosis, drug or alcohol abuse with or without dependence.

**Figure S3: Risk factors of repeat ablation following catheter ablation of atrial fibrillation**

**
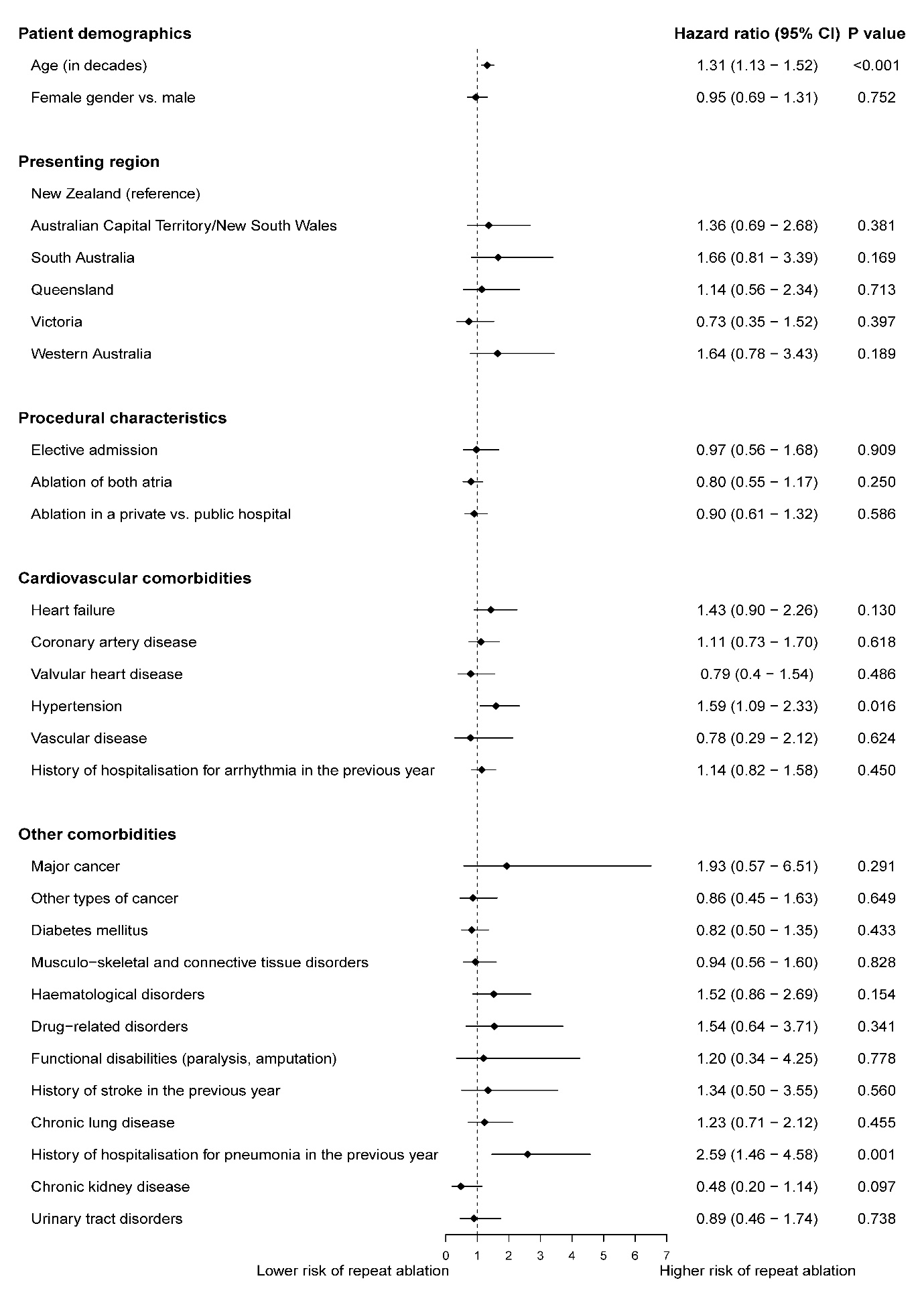
**

**Footnote:** Major cancer includes metastatic cancer and acute leukemia; lung, upper digestive tract, and other severe cancers; and lymphatic, head and neck, brain, and other major cancers. Drug-related disorders includes drug or alcohol psychosis, drug or alcohol abuse with or without dependence.
